# Synthesis of Biobased and Hybrid Polyurethane Xerogels from Bacterial Polyester for Potential Biomedical Applications

**DOI:** 10.3390/polym13234256

**Published:** 2021-12-04

**Authors:** Sophie Wendels, Deyvid de Souza Porto, Luc Avérous

**Affiliations:** BioTeam/ICPEES-ECPM, UMR CNRS 7515, University of Strasbourg, 25 Rue Becquerel, 67087 Strasbourg, France; sophie.wendels@etu.unistra.fr (S.W.); deyvid.porto@gmail.com (D.d.S.P.)

**Keywords:** biobased, xerogel, organic-inorganic network, polyurethane, fatty-acid derived isocyanate

## Abstract

Organic–inorganic xerogel networks were synthesized from bacterial poly (3-hydroxybutyrate) (PHB) for potential biomedical applications. Since silane-based networks usually demonstrate increased biocompatibility and mechanical properties, siloxane groups have been added onto polyurethane (PU) architectures. In this work, a diol oligomer (oligoPHB-diol) was first prepared from bacterial poly(3-hydroxybutyrate) (PHB) with an environmentally friendly method. Then, hexamethylene diisocyanate or biobased dimeryl diisocyanate was used as diisocyanate to react with the short oligoPHB-diol for the synthesis of different NCO-terminated PU systems in a bulk process and without catalyst. Various PU systems containing increasing NCO/OH molar ratios were prepared. Siloxane precursors were then obtained after reaction of the NCO-terminated PUs with (3-aminopropyl)triethoxysilane, resulting in silane-terminated polymers. These structures were confirmed by different analytical techniques. Finally, four series of xerogels were prepared via a sol–gel process from the siloxane precursors, and their properties were evaluated depending on varying parameters such as the inorganic network crosslinking density. The final xerogels exhibited adequate properties in connection with biomedical applications such as a high in vitro degradation up to 15 wt% after 12 weeks.

## 1. Introduction

Polyurethanes (PUs) are one of the main polymer families, with a ranking of 6th amongst all polymers [1] due to their wide range of applications, easy synthesis and great possibility to adapt their properties by varying their formulation. In addition, PUs stand out for their mechanical, thermal and chemical properties, to be used as, e.g., adhesives, foams or coatings for a large range of applications, including biomedical applications [2,3,4,5].

Within the perspectives in obtaining polymers with low environmental impact, the development of new macromolecular architectures from renewable resources has generated considerable attention in all domains since the last decades [6,7,8]. Many different sources of biomass are nowadays largely available, like lipids (vegetable oils) [9,10] or polysaccharides (lignocellulosic derivatives, alginate, chitin, etc.) [11,12,13], for instance. White biotechnology, which is a branch of biotechnology using living organisms for the synthesis of new molecules and macromolecules, is also largely developed to obtain different building blocks (short molecules), macromers, or bacterial biopolymers such as Polyhydroxyalkanoates (PHA), a family of biopolyesters [6,14]. From these molecules and macromolecules, new architectures can be synthesized and tailored for specific applications. However, the obtained systems and products need to exhibit adequate and new properties in order to be competitive with fossil-based polymers. To obtain innovative and tunable properties, grafting [15,16], blending, composites or the insertion of inorganic elements into the macromolecular chains [17,18] are explored to fulfill the challenging requirements.

**Scheme 1:**
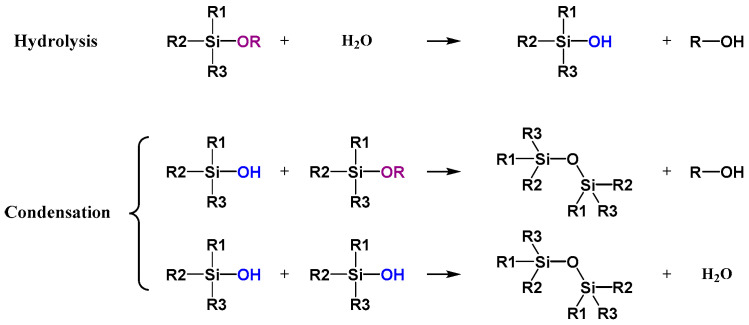
Hydrolysis and condensation chemical reactions involved in the sol–gel process.

In order to improve PUs performances, and more specifically for biomedical applications [5], hybrid organic–inorganic systems based on siloxane networks have recently emerged as promising candidates. The introduction of silanes into the PU backbone can improve the final properties of the material such as thermal stability and tensile strength, for instance [19,20]. The main synthetic route to silane-modified PU consists of the incorporation of silane precursors in a PU matrix, followed by a sol–gel process in situ (Scheme 1) [21,22,23]. The obtained composite contains inorganic SiO_2_ nanoparticles (NPs). Another way is the preparation of chemically linked organic–inorganic networks. Their synthesis usually lies in an isocyanate (NCO)-terminated PU prepolymer (PUP), which reacts with primary or secondary aminosilanes, forming urea bonds [24,25]. Depending on the type of aminosilane used [4], the formation of, e.g., hydrogen-bonded urea monodentate or bidentate is known to yield higher physical and mechanical performances. The synthesis of silane-terminated PU (SPU) can be prepared in bulk or in aprotic solvent to avoid reactions with free NCO, for instance. Then, to form the inorganic matrix, the SPU acts as siloxane precursor and will undergo a sol–gel transition in a chosen solvent, as described in Scheme 1. In general, the “sol” is formed by hydrolysis and condensation of metallic or metalloid alkoxides (e.g., Si-based) in the presence of water, forming a colloidal suspension. With the increase of the condensation reactions in silane alkoxides, a 3D-network structure with Si-O-Si bonds is formed, called “wet gel”. After conventional drying, a xerogel is obtained. By exchange of solvent for supercritical CO_2_, hence maintaining the pores and the structural network, the final material is called “aerogel” [26,27]. Silane-modified PUs have been evaluated as performing materials due to their tunable physico-chemical properties, low or non-toxicity and biocompatibility [28,29]. For that, they have been explored for several biomedical applications such as wound dressing [30], scaffold [31] or as biocompatibility enhancer [23].

**Scheme 2:**
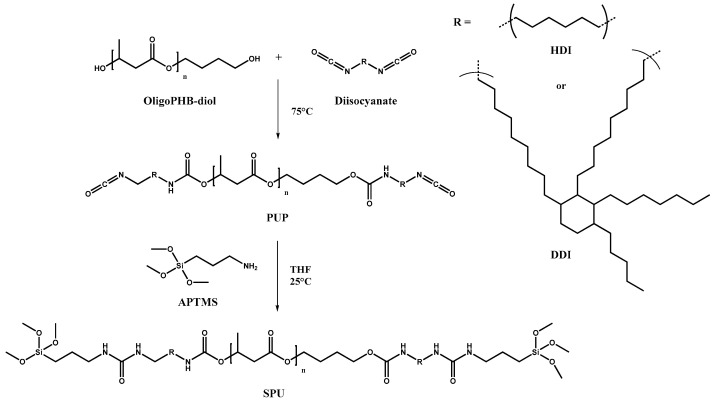
Synthetic pathway for the synthesis of the SPUs.

The formation and evaluation of renewable systems like PHA-based SPU xerogels have been rarely reported. In this way and in this work, poly(3-hydroxybutyrate) (PHB) obtained by fermentation of sugar biomass was used in the synthesis of different series of xerogels. SPUs architectures were prepared from previously synthesized short PHB diol oligomers (oligoPHB-diol) with a diisocyanate, and 3-aminopropyltrimethoxysilane (APTMS) as a sol–gel precursor. To obtain various chemical architectures, two different diisocyanates have been used: a fossil-based, hexamethylene diisocyanate (HDI), and biobased dimeryl diisocyanate (DDI) (Scheme 2). This latter, obtained from fatty acids dimerization, presents aliphatic pending chains, which bring local mobility [32]. To vary the inorganic crosslinking density, SPUs were also partially replaced by tetraethyl orthosilicate (TEOS) in some compositions. In total, four series of xerogels films were obtained (Table 1). Their thermal, mechanical and physico-chemical behaviors were mainly evaluated in connection with potential biomedical applications.

## 2. Experimental Section

### 2.1. Materials

PHB with a mass-average molar mass (M_w_) of 520,000 g.mol^−1^ and Dispersity (Ð) close to 2, of trade name PHB L88, was kindly supplied by Biocycle, Brazil. This bacterial biopolyester was obtained from microbial fermentation with *Cupriavidus necator* from sugarcane, after extraction and purification. It was kindly supplied by Biocycle (Brazil). PHB was dried under vacuum in an oven at 40 °C overnight before use. DDI (Scheme 2), a biobased dimer fatty-acid derived diisocyanate, was kindly offered by Cognis-BASF. 1,4-butanediol (BDO) (99%) was purchased by Alfa Aesar. Dibutyltin dilaurate (DBTL) (95%), deuterated chloroform (CDCl_3_), HDI (>99%), dibutylamine (99.5%), phosphate buffer saline (PBS) tablets were purchased from Sigma-Aldrich. Hydrochloric acid (HCl), ethanol, chloroform (>98%) were obtained from VWR. Petroleum ether and tetrahydrofuran (THF, extra pure, stabilized with 0.025 BHT) were purchased from Fisher Chemical, and TEOS (99%) and APTMS (97%) were obtained from Fluorochem.

### 2.2. OligoPHB-Diol Synthesis

The oligoPHB-diol synthesis was performed using a previously described method [32]. For that, BDO (6000 molar equivalent) was heated up under argon flux to 180 °C with an oil bath. Dried PHB (1 molar equivalent) was added and the mixture was magnetically stirred until PHB dissolution (~1 h). DBTL (2.2 molar equivalent) was then added to the reaction mixture. After a precise and predetermined reaction time related to the final oligomer molar mass [32], the reaction mixture was precipitated and washed three times with large volumes of petroleum ether to extract the catalyst. After removing the petroleum ether, the precipitate was further treated with a mixture of chloroform and water (1/1 volume per volume *v*/*v*) to remove the BDO excess by liquid–liquid separation. The chloroform phase containing the oligomers was finally distilled under reduced pressure to yield 300 g.mol^−1^ oligoPHB-diols. The oligomers were kept in an oven at 40 °C under vacuum for 16 h.

### 2.3. Synthesis of PUPs

OligoPHB-diols (M_n_ = 300 g/mol) were reacted with HDI or DDI using different NCO/OH molar ratios (2/1 for DDI, and 2/1, 4/1, 6/1 for HDI, respectively) in a bulk process without catalyst to fulfill different green chemical principles. For that, HDI or DDI was introduced into a flame-dried three-neck round-bottom flask and heated with an oil bath at 80 °C under argon flux and mechanical stirring. Then, oligoPHB-diol was added to the reaction mixture. Aliquots were withdrawn at different reaction times to monitor the reaction extent by back titration to obtain the free NCO content (%NCO), following standard protocols [33]. The reactions were stopped after the theoretical %NCO was obtained, and then stored under argon. They are named as PUP2-1, PUP4-1 and PUP6-1 for HDI and the 2-1, 4/1 and 6/1 NCO/OH molar ratio, respectively. The DDI-based system was named PUPD2-1 (Table 1).

### 2.4. Synthesis of SPUs

In a two-neck round-bottom flask, the PUP was added in dry THF at a ratio 2/1 mass per volume (*m*/*v*, *m* in g and *v* in mL) and was magnetically stirred under an argon-flux for 5 min. Then, a precise amount of APTMS was added into the mixture to yield silane end-capped PUs with a NCO/NH_2_ molar ratio of 1 (Table 1). For that, a precise solution amount of APTMS in dry THF at a ratio 2/1 (*m*/*v*) was added dropwise and the system was stirred at room temperature for 25–30 min. THF was then exchanged with dry ethanol by distillation at 80 °C to yield SPU. They were named SPU2-1, SPU4-1, SPU6-1 and SPUD2-1 and were synthesized from PUP2-1, PUP4-1, PUP6-1 and PUPD2-1, respectively.

### 2.5. Synthesis of Xerogel from SPU

The sol–gel process was started from the addition of water to the SPU solutions in dry ethanol. Several ratio alkoxide group (OMe)/H_2_O (R_w_) of 1/1, 2/1 and 8/1 were chosen. After addition, the solutions were magnetically stirred for 1 min. The mixture was poured in a Teflon mold, covered with parafilm^®^ and maintained at room temperature for 24 h to allow hydrolysis and condensation reactions. Then, wet gels were dried in an oven at 40 °C for 48 h, followed by 24 h at 60 °C until complete evaporation of ethanol and possible residues of THF. Xerogel films were obtained with 1–2 mm thickness.

In total, 8 xerogels were obtained and can be classified into four different series (Table 1). Each series corresponds to the variation of one parameter. The four main series are based on:

(1) A variation of NCO/OH molar ratio in the PUP (2/1, 4/1 and 6/1), with R_w_ = 2, giving three xerogels–polymer hybrids named XP2-1R2, XP4-1R2 and XP6-1R2, respectively. A higher NCO/OH molar ratio yield SPU with greater APTMS amount and thus xerogels with higher inorganic networks.
(2) A variation of R_w_ (1/1, 2/1 and 8/1). This series was prepared from SPU2-1 and the hybrid materials were called XP2-1R1, XP2-1R2 and XP2-1R8. Reducing R_w_, hence increasing the water content, decreases the condensations, but promotes hydrolysis reactions in the sol–gel process. However, is it supposed that R_w_ = 1/1 leads to almost or complete hydrolysis and thus very low or no condensation [27].
(3) The variation of the diisocyanate used in the SPU synthesis: HDI for SPU2-1 and DDI for SPUD2-1, with R_w_ = 2, giving two different xerogels named XP2-1R2 and XPD2-1R2, respectively. Contrary to HDI, DDI is composed of long aliphatic pending chains, which could bring more mobility and flexibility to the final xerogel.
(4) The gradual replacement of SPU by TEOS using TEOS/SPU ratio of 0, 1/3 and 1/1 and R_w_ = 2. The corresponding xerogels were named XP2-1R2, XPT3-1 and XPT1-1, respectively. Contrary to APTMS, which has an organic chain and amine end-group chemically linked to the xerogels PU, the incorporation of fully inorganic TEOS promotes hyperbranched Si-O-Si networks with no interaction to the PU.

### 2.6. General Methods and Analysis

Xerogels biobased carbon contents (in wt%) were evaluated by calculation from the starting biobased materials as follows: OligoPHB-diols and DDI can be considered containing 100 and 98% biobased carbon, respectively, according to ASTM-D6866-12 Method B (AMS) standard procedure [34].

The %NCO was obtained by an indirect titration method. For that, DBA (50 mL of a 0.2 M solution in dry THF) was added to a precise mass of PUP (1–2 g) and reacted for 2 min. The resulting amine excess was then titrated using a standard aqueous HCl 0.5 M solution and bromophenol blue as indicator. The free NCO content, given in weight per cent, was calculated by Equation (1), where V_b_ (mL) is the HCl solution volume necessary for the blank titration, vs. (mL) the HCl solution volume required for the sample titration, and M (g) the prepolymer weight:

(1) $$\%\mathrm{NCO}=\frac{\left(\mathrm{Vb}-\mathrm{Vs}\right)\times 4202\times 0.5}{M\times 1000}$$

^1^H NMR spectra were obtained with a Bruker 400 MHz spectrophotometer using CDCl_3_ as deuterated solvent. The number of scans was set to 16. The CDCl_3_ peak (*δ*_H_ = 7.26 ppm) was used as reference.

Fourier transformed infrared spectroscopy (FTIR) was performed on a Nicolet 380 spectrometer (Thermo Electron Corporation) used in reflection mode and equipped with an attenuated total reflectance (ATR) diamond module, also called FTIR-ATR. The spectra were obtained at a 4 cm^−1^ resolution and with 32 scans.

Thermogravimetric analysis (TGA) was performed under nitrogen atmosphere at 25 mL.min^−1^ using a TGA Q5000 apparatus from TA instruments. Samples (1–3 mg) were heated from room temperature up to 650 °C at a rate of 10 °C/min.

Differential scanning calorimetry (DSC) was performed using a TA Instrument Q200. Samples (2–3 mg) were placed in sealed aluminum pans and analyzed under nitrogen flow (50 mL/min). A three-step procedure with a 10 °C/min ramp was applied as follows: (1) heating up from room temperature to 185 °C and holding for 3 min to erase the thermal history, (2) cooling down to −80 °C and holding for 3 min, (3) heating (second heating) from −80 °C to 200 °C.

Dynamic mechanical analyses (DMA) were analyzed using a TA Instruments Discovery Hybrid Rheometer HR-3 in dynamic mode. The rheometer was equipped with rectangular torsion geometry. Temperature range varied from −50 °C to 150 °C at 2 °C/min, a 1 Hz frequency and a strain of 0.01%. Crosslink density (*ν_e_*) and the molar masses between the crosslinking points (M_c_) were obtained using Equations (2) and (3), respectively [35,36]:

(2) $$\nu_{e}=\frac{E^{\prime }}{3RT}$$

(3) $$M_{c}=\frac{\rho }{\nu_{e}}$$

where *E′* is the storage modulus taken in the rubbery plateau at *T* = T_g_ + 85 °C, R is the universal constant of gases (8.314 J.mol^−1^.K^−1^), *T* is the temperature in kelvin (K) and *ρ* is the density of the xerogels. ρ values were measured as the mean values from sets of four 1 cm discs with ~1 mm thickness. The mean ρ value for all xerogels was 1.47 g.cm^−1^.

Uniaxial tensile tests were performed on an Instron 5567H (USA) with a 10 kN load cell. The experiments were carried out at room temperature with a constant crosshead speed of 20 mm.min^−1^. Sets of five dumbbell-shaped samples with dimensions of approximately 45 × 5 × 1 mm^3^ were cut tested. Young’s modulus, tensile strength at break (σ_max_) and elongation at break (ε_max_) were calculated from the curves. XP6-1R2 was too brittle to be cut in dumbbell shapes.

The apparent water contact angle (WCA) was determined using a DSA25 goniometer (Krüss). For that, 10 drops of deionized water (6–8 µL) were deposited on the materials surfaces. Pictures were taken after the drop stabilization (~10 s). The WCA were calculated, and the obtained results are given as mean values with standard deviations and a representative picture.

In vitro degradation of 1 cm (~100 mg) diameter xerogel discs was carried out in analogy to ASTM F1635-04 standard. Dried discs were weighed (*m*(0)) and immersed in 5 mL of 50 mM PBS solution at pH 7.4 and 37 °C. At predetermined time points (1, 2, 3, 4, 8 and 12 weeks), samples were recovered, rinsed with distilled water, and dried for at least 24 h under vacuum in an oven at 30 °C. The degradation medium was changed at each time point. For each xerogel five repetitions were performed. The weight loss was calculated using Equation (4):

(4) $$\mathrm{Weight}\;\mathrm{loss}\;\left(\%\right)=\frac{m\left(0\right)-m\left(t\right)}{m\left(0\right)}\times 100$$

where *m*(*t*) is the dry sample weight at predetermined time *t*.

Scanning electronic microscopy (SEM) was used to study the evolution of the surface morphologies of non-degraded and degraded xerogel films after exposure to hydrolytic degradation. A Tescan Vega-3 apparatus in low vacuum mode (20 Pa, LVSTD and Univac) with an acceleration voltage of 20 kV and working distances in the range of 10–12 mm was used. Before examination, samples were coated with a thin layer of gold using a sputter coater (Quorum Q 150 RS, Quorum Technologies, Laughton, United Kingdom).

## 3. Results and Discussion

### 3.1. Xerogels Synthesis

**Figure 1:**
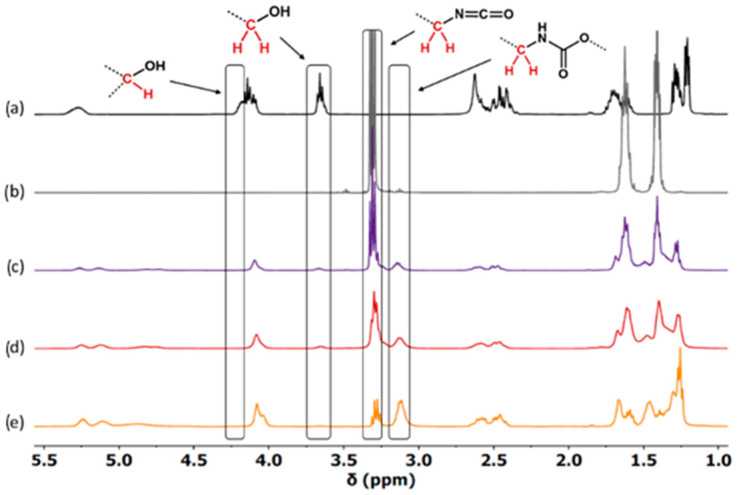
^1^H-NMR Spectra for OligoPHB-diols and the different PUPs. From top to bottom: (**a**) oligoPHB-diol, (**b**) HDI, (**c**) PUP6-1, (**d**) PUP4-1 and (**e**) PUP2-1. Main functions and hydrogens are depicted on top of the figure, and corresponding changes in the spectra are circled.

^1^H-NMR spectra of the oligoPHB-diol is presented in Figure 1. Characteristic signals at δ = 5.29 and 2.44–2.65 ppm were attributed to -CH(CH_3_)-CH_2_-CO- and -CH(CH_3_)-CH_2_-CO- protons from the PHB repetitive unit, respectively, while chemical shifts at δ = 3.67 and 4.19 ppm were assigned to protons from primary and secondary hydroxyl, respectively. Several additional physico-chemical and thermal properties were given in a previous publication [32].

The chemical structure of the four PUPs was confirmed by ^1^H-NMR. Spectra are presented in Figure 1 for the HDI-based PUP and in Supporting Information (Figure SI.1) for PUPD2-1 compared to neat DDI and oligoPHB-diol. Protons from oligoPHB-diol hydroxyl end functions (δ = 3.67 and 4.19 ppm) disappeared after reaction with -NCO groups to form urethane bonds. Chemical shift at δ = 3.14 ppm was assigned to -CH_2_-NH-CO-O protons from HDI or DDI, and at δ = 3.29 ppm to protons adjacent to the terminal -NCO groups. Finally, signals in the region δ = 1.57–1.77 ppm correspond to the superimposition of -CH_2_- protons from oligoPHB-diol, HDI and DDI chains. From PUP2-1 to PUP6-1, the peak at chemical shift δ = 3.29 ppm had increasing intensity due to the presence of HDI free monomers in higher amounts in PUP4-1 and PUP6-1, and thus of terminal -NCO. On the contrary, a small peak was observed when using DDI, mainly in comparison to the high intensity of the -CH_2_- protons peak region (δ = 1.57–1.77 ppm) coming from its aliphatic pending chains (Scheme 2).

**Figure 2:**
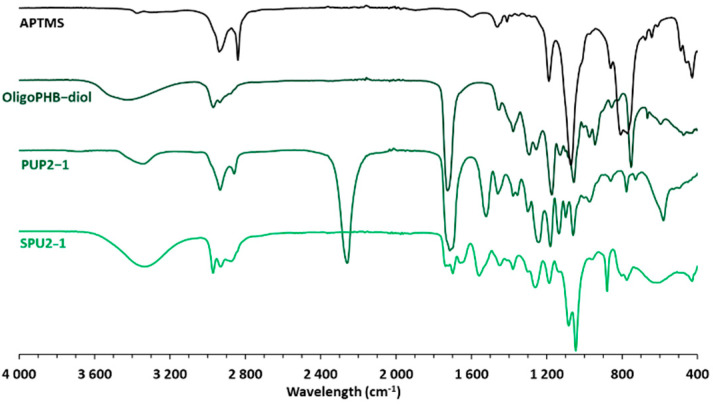
FTIR spectra of the synthetic route (from top to bottom), from APTMS and oligoPHB-diol to SPU polymer, for SPU2-1.

For the xerogels multistep synthesis, the structure of the different intermediate systems oligoPHB-diol, PUP and SPU (Scheme 2) were analyzed by ATR-FTIR. Figure 2 shows the spectrum of APTMS, the oligoPHB-diol, PUP and SPU for the synthesis of SPU2-1. In the first step of reaction from oligoPHB-diol to PUP, the disappearance of the O-H stretching vibration from the oligoPHB-diol reaction with HDI was shown at 3400 cm^−1^, as well as the appearance of characteristic NCO-terminated PU bands in PUP at 3329, 2260, 1700 and 1515 cm^−1^. They were attributed to the urethane stretching vibrations from N-H, N=C=O from terminal functions and C=O bonds, and urethane N-H bending vibrations, respectively.

In the second reaction step from PUP2-1 to SPU2-1, the complete reaction between NCO end-groups and APTMS was confirmed by the disappearance of the N=C=O stretching vibration band at 2260 cm^−1^, as well as the appearance of a sharp peak at 1678 cm^−1^. It was attributed to urea bonds obtained by terminal NCO reaction with primary amine groups from APTMS. Characteristic bands of the SPU alkoxysilane were assigned in the range 1000–1100 cm^−1^ and correspond to Si-O-CH_3_ bonds, probably mixed with some silanol end-groups (Si-OH), which was confirmed by the presence of the peak broadening at 3330 cm^−1^.

### 3.2. Analysis of the Hybrid Organic-Inorganic Xerogels

**Figure 3:**
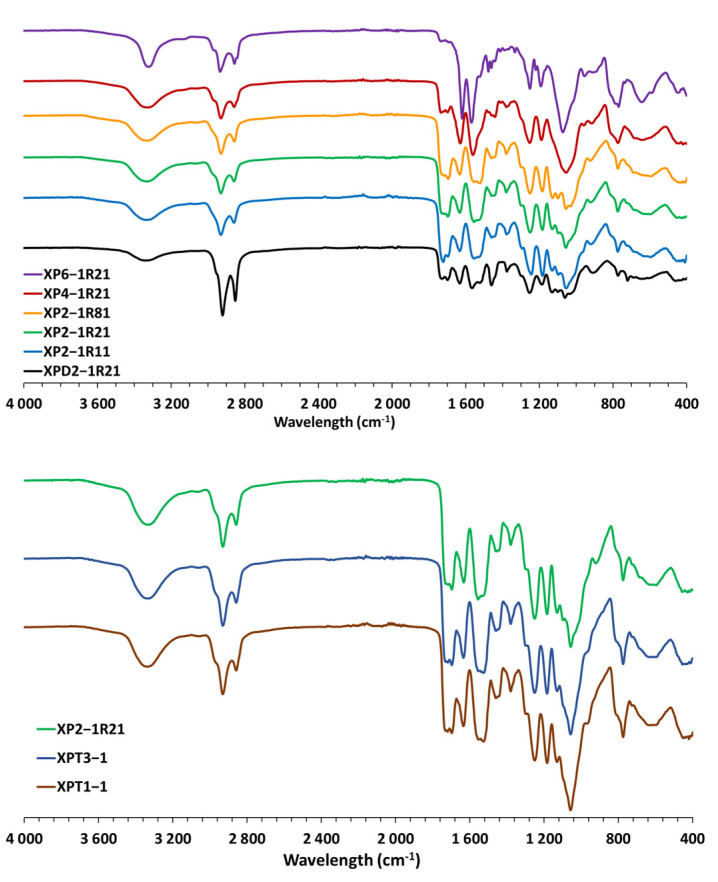
Xerogels FTIR-ATR spectra.

FTIR-ATR analyses of the hybrid xerogels were finally carried out. Spectra are presented in Figure 3. Characteristic bands of the PU backbone at 3320, 1615 and 1240 cm^−1^ were attributed to the N-H stretching vibration, N-H bending vibration and C-N stretching vibration from urethane/urea bonds, respectively. The broad peak at 1715–1730 cm^−1^ corresponds to the superposition of C=O stretching vibrations from urethane and urea, as well as esters from oligoPHB-diol. Moreover, the sharp band at 1180 cm^−1^ was assigned to the C-O stretching vibration due to the oligoPHB-diol building blocks in the global architecture. Inorganic network was evidenced by the apparition of the broad peak at 1000–1150 cm^−1^, which corresponds to the superposition of the Si-O-Si, C-Si-O and Si-O-C absorption bands, as well as by the Si-O-Si rocking vibration at 445 cm^−1^. Incomplete sol–gel condensation reactions, hindered from high water amount/low R_w_ (Series 2) [27], leads to hydrogen-bonded Si-OH which were visible in the 3300–3370 cm^−1^ region with a peak broadening, and at 950–920 cm^−1^ in form of a broad, single band. With the increase of the NCO/OH ratio in Series 1, a bigger Si-O-Si inorganic network was formed due to the higher APTMS content in (Table 1). This resulted in an intensification of the corresponding bands at 1000–1150, 445, 3300–3370 and 950–920 cm^−1^. Moreover, the same conclusion can be drawn from the addition of increasing amounts of TEOS into the xerogel (Series 4) with specific and more intense bands of the siloxane inorganic network.

**Figure 4:**
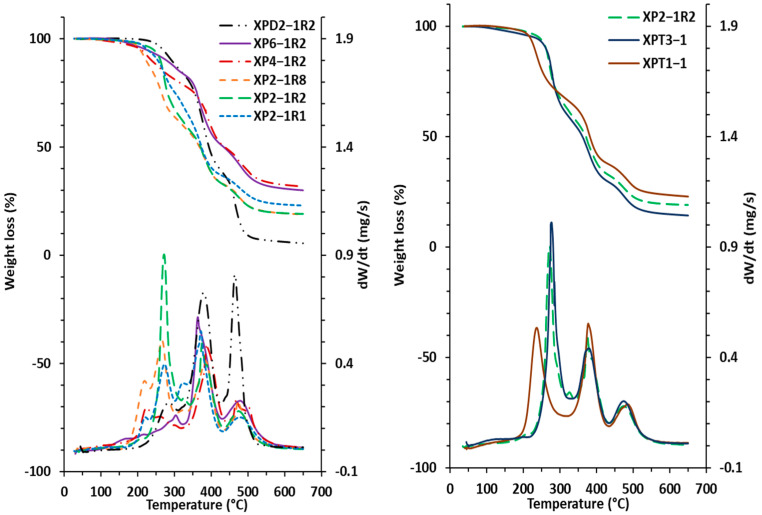
TGA (top) and DTG (bottom) spectra of the synthesized xerogels, describing the weight loss and derivate as a function of the temperature.

The xerogels thermal stability analyses, displayed in Figure 4, were carried out by TGA in nitrogen from room temperature to 650 °C. Taking the onset degradation temperature (T_5%_), all gels exhibited good thermal stability, as no T_5%_ was recorded before 200 °C. The degradation onset is usually ascribed to the less thermally stable part of the material, which correspond to the organic PU urethane bonds in the xerogels [37,38], as well as oligoPHB-diol esters as previously described [32]. A similar trend was observed for all formulations, as the degradation happened in three main steps, from which the maximum degradation rate temperatures are represented by T_deg1,max_, T_deg2,max_ and T_deg3,max_ in Table 2. The first degradation step at 200–300 °C, where T_5%_ is found, was assigned both to the PUs urethane bonds and oligoPHB-diol esters degradation. The second degradation step, occurring at 350–390 °C and represented by T_deg2,max_, was ascribed to organic chains scissions of oligoPHB-diol-, HDI-, DDI- and APTMS-based building blocks in the global architectures. The last degradation step (>450 °C) was assigned both to the loss of remained PU residues and to the inorganic segment. Thus, with XPD2-1R2 containing long aliphatic grafted chains from DDI (Scheme 2), the mass losses given by T_deg2,max_ and T_deg3,max_ were significantly higher than for short HDI-based xerogels. Comparing the different HDI-based xerogels, one can confirm the increase in thermal stability coming from the siloxane network. In fact, gels with higher NCO/OH ratios (Series 1), lower R_w_ (Series 2) and increasing TEOS content (Series 4) exhibited T_deg3,max_ shifts to higher temperatures, due to a higher Si-O-Si network [39]. Another interesting indicator of the xerogel thermal stability is the char residue linked to the inorganic network, which could be in part linked to intrinsic flame-retardancy properties of silica-based materials by intumescence [40]. In fact, char residues content at 650 °C are directly linked to the formed siloxane network [41]. As expected, XPD2-1R2 exhibited the lowest char yield due to the high DDI molar mass compared to HDI, resulting in low APTMS content and thus siloxane network. By comparing the increase in R_w_, XP2-1R2 and XP2-1R8 exhibited similar properties with a char residue of around 19%, while there was a slight increase in the char residue of XP2-1R1 due to larger condensation reactions during the xerogels drying process. Unsurprisingly, char residue was significantly higher with higher NCO/OH molar ratios. The APTMS molar equivalent increase (Table 1) resulted in a consequent addition of siloxane precursor Si-(OMe)_3_ in the material, and thus in an expanded inorganic network content. The partial replacement of SPU by TEOS did not have a significant impact on the TGA results, except for XTP1-1 (highest TEOS content). The addition of TEOS results in a lower organic phase, as it is purely inorganic compared to APTMS. Hence, with the incorporation of equimolar amount of SPU and TEOS, XPT1-1 resulted in a lower T_5%_ and a greater char residue compared to XPT3-1 and XP2-1R2.

TGA results have shown that in the DSC temperature range, no major degradation occurs. DSC analyses were performed to study the effect of the organic–inorganic network on the T_g_ of the materials. The spectra are available in Supporting Information (Figure SI.2). The observed T_g_ (Table 2) correspond to the organic PU transitions. First, XPD2-1R2 had a significantly lower T_g_ than the other xerogels due to the presence of flexible pending chains from the DDI-based building blocks with a softening effect. This behavior is typical with DDI and can be compared to HDI [32]. After comparison of the HDI-based xerogels, there was no significant difference between the T_g_, which were comprised between 9 and 20 °C. The highest T_g_ was observed for XP6-1R2, probably due to the presence of more urea bonds from the APTMS amine end-group reaction with NCO.

**Figure 5:**
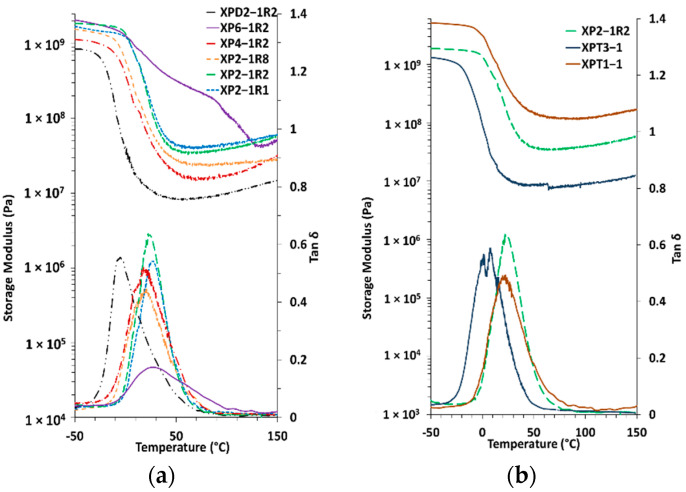
DMA spectra of the organic–inorganic networks. Storage modulus and tan δ evolutions are given in the top and the bottom of the spectra, respectively. (**a**) corresponds to Series 1, 2, 3 and (**b**) to Series 4.

Figure 5 exhibits the dynamic mechanical behavior of the synthesized xerogels by DMA, to evaluate the viscoelastic properties and the crosslinking densities. Each material showed one major transition associated to their relaxation temperature (T_α_). This transition was characterized by a strong decrease in E’ coupled with a peak in the tan δ curve. The T_α_ determined at the maximum of the tan δ peak are summarized in Table 2. T_α_ at 1 Hz can be linked to the T_g_ obtained by DSC [42] and are easily determined since the peaks are rather narrow. Obtained T_α_ values were in good accordance with T_g_ results previously obtained. With NCO/OH ratio increase, there was first a slight decrease of T_α_ from XP2-1R2 to XP4-1R2 (22 to 18 °C), followed by an increase to 28 °C for XP6-1R2. The same tendency was already observed by DSC results (Table 2). T_g_ and T_α_ were expected to increase from XP2-1R2 to XP6-1R2, due to higher physical interactions from urea. An equivalent phenomenon was observed between XP2-1R1, XPT3-1 and XPT1-1, where a gradual increase of T_g_ and T_α_ was also obtained. These results might be explained by a lower crosslinking density in the sol–gel process. Condensation reactions being highly dependent on several parameters such as the solvent, the temperature or relative/absolute content on precursor mixtures, for instance [27], small variations in these parameters can have a significant effect on the final network.

All tan δ peaks showed similar shapes. They are rather narrow with quite low intensities. Usually, the peak intensity is linked to the amorphous phase of the material. For instance, XP6-1R2 exhibited the lowest peak intensity linked to higher PU physical interactions from urea, urethane and ester bonds compared to the other xerogels. Regarding the broadness, it is connected to the homogeneity of the PU chains dispersity. All materials exhibited similar tan δ peak broadness. Then, the PU phase dispersity should be similar in all xerogels. At higher temperatures (T > 50 °C), E’ curves exhibited the formation of a rubbery plateau for all samples, which is characteristic of crosslinked materials. In order to further investigate these properties, crosslink densities and molar masses between the crosslinking points were determined from the curves using Equations (2) and (3). Main values are summarized in Table 2. As expected, the crosslinking increased (i) with a lower R_w_, from 2680 to 4900 mol.m^−3^ for XP2-1R8 and XP2-1R1, respectively. It also increased (ii) with a higher NCO/OH ratio, respectively, with 4269 and 8508 mol.m^−3^ for XP2-1R2 and XP6-1R2, and (iii) by replacing SPU by TEOS. The results also confirmed the lower crosslink densities of XP4-1R2 and XPT3-1R2 which explained the decreased T_g_ and T_α_ observed earlier in DMA and DSC.

**Figure 6:**
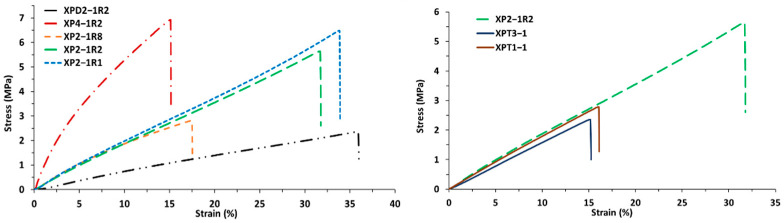
Uniaxial tensile test. Stress–strain curves for the different xerogels.

Uniaxial tensile tests were performed to analyze the static mechanical properties of the xerogels. The curves are displayed in Figure 6 and corresponding data are summarized in Supporting Information (Table SI.1). All xerogels based on the SPU2-1 polymer exhibited similar Young’s moduli, at around 19 ± 3 MPa. When comparing the xerogels by Serie, the Young’s moduli seemed mainly dependent on the organic PU content and architecture, and not on the inorganic crosslinking density. Moreover, in Series 1 when the organic phase was increased from XP2-1R2 to XP4-1R2, the elastic modulus significantly increased from 21 to 79 MPa, probably from the higher organic content and urea bonds forming physical interactions. Here, XP6-1R2 was not tested since it was too fragile to properly obtain dumbbells. However, one could assume that it had a higher modulus than XP4-1R2. As expected, the xerogels with the lowest modulus was XPD2-1R2 due the DDI building blocks, which is known to form flexible materials with lower moduli than conventional diisocyanates [32,34]. Compared to the Young’s modulus, the tensile strength and elongation at break were influenced by the inorganic crosslinking density of the gels. Tensile strength at break increased with the decrease of R_w_ (Series 2) and the NCO/OH ratio (Series 1) because of a greater crosslinked inorganic network formed (See Table 2). With the SPU replacement by TEOS building blocks (Series 4, from XP2-1R2 to XPT3-1), the neat inorganic network was enhanced to the detriment of the organic-inorganic network, inducing higher brittleness and lower tensile strength and elongations at break. Finally, comparing the diisocyanate used (Series 3), XPD2-1R2 displayed the longest elongation at break and lowest tensile strength amongst all xerogels prepared, due to the long and flexible DDI chains.

**Figure 7:**
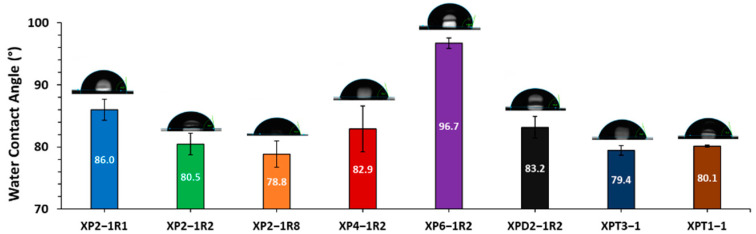
Xerogel materials with the average water contact angles, standard deviations and representative pictures.

Figure 7 exhibits the measured sessile WCA of the xerogels, with the average values and representative pictures of the drops shapes. Most xerogels displayed slightly hydrophilic behavior with contact angles < 90°, except XP6-1R2, which was slightly hydrophobic (96.7°). As expected, increasing the crosslinked siloxane network content (Table 2) leads to a significant increase of the apparent contact angle due to a hydrophobic Si-O-Si network, as already described in previous studies [23,43]. Contact angles indeed increased from 78.8 to 86.0° with decreasing R_w_, and from 78.8 up to 96.7° for XP2-1R2 and XP6-1R2 in Series 1, respectively. A slight but not significant increase in contact angle was observed between the HDI- and the DDI-based xerogels due to the intrinsic higher hydrophobicity of DDI and its long aliphatic grafted chains. Finally, one could have expected an increase of contact angle with the gradual substitution of SPU polymer by TEOS, hence between XP2-1R2, XPT3-1 and XPT1-1. However, the measured angles were similar, at around 80°.

**Figure 8:**
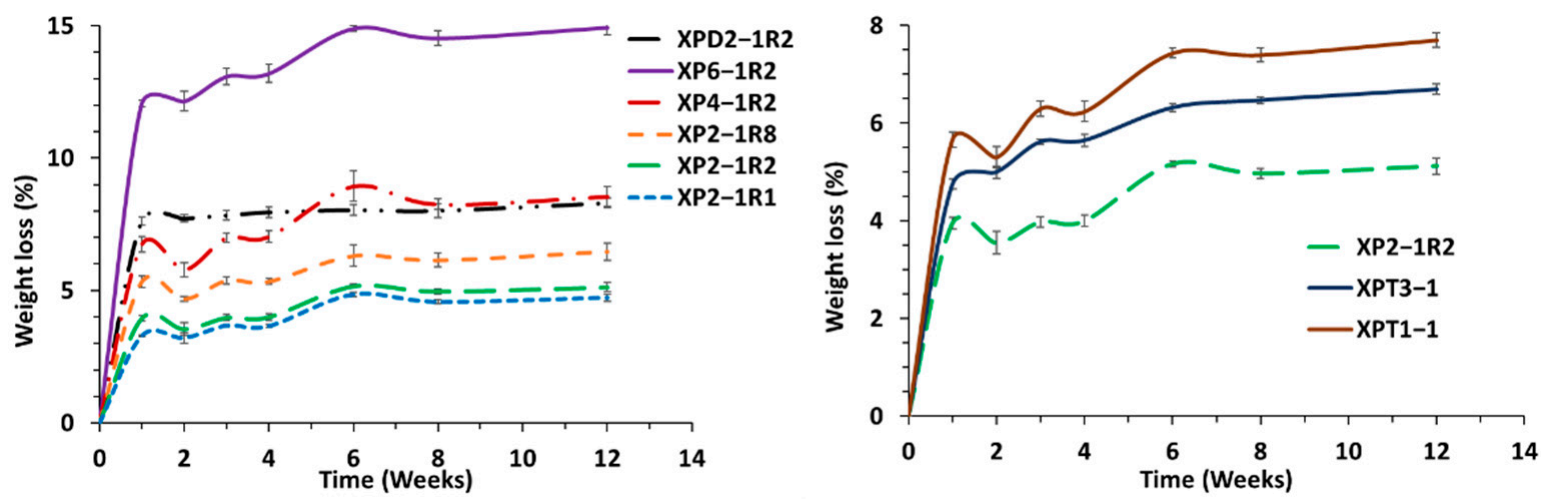
Evolution of the xerogels degradations (weight loss in %) in PBS at 37 °C for 12 weeks.

In vitro degradation of the xerogels in PBS at 37 °C was performed over a 12 weeks-period in order to start their evaluation for specific biomedical applications. Main results are displayed in Figure 8 with standard deviations. All xerogels exhibited similar behavior with a significant weight loss after the first week of incubation, followed by lower but constant decrease over time. Hydrolytic degradation of siloxane-based materials such as polydimethylsiloxane (PDMS) has been extensively studied in various conditions, mainly for biomedical applications such as implants [44]. In our case, the degradation is mainly driven by the inorganic content. The low amount of hydrolysable bonds such as esters from oligoPHB-diol is the main responsible for the poor weight losses from organic content degradation. Here, Si-O-Si bonds lead to hydrophobic surfaces, as described earlier in the WCA analysis in Figure 7. However, they are sensible to hydrolytic degradation with the formation of silanol groups Si-OH, as depicted in Supporting Information (Scheme SI.1) [27]. Hence, in highly inorganic networks with low molar masses between crosslinking points, short silanol-terminated molecules can be obtained from the hydrolytic degradation of Si-O-Si bonds. Silanol groups bring a high hydrophilicity compared to hydrophobic Si-O-Si. Then, these short molecules tend to migrate into the PBS solution, resulting in the materials weight loss. In organic–inorganic networks, the weight loss is thus directly linked to the molar mass between siloxane groups. For instance, a significant increase in weight loss is linked with the NCO/OH ratio (Series 1). They were of 5.1, 8.6 and 14.9 wt% for XP2-1R2, XP4-1R2 and XP6-1R2, respectively. This increase was first due to the higher inorganic crosslinking density (Table 2). Secondly, it was due to the presence of low molar mass SPUs from the APTMS reaction with the excess of HDI in PU4-1 and PU6-1, forming *in fine* short labile silanol-terminated molecules from Si-O-Si hydrolytic degradation, finally increasing the weight loss. Surprisingly, there was a higher weight loss with XPD2-1 (8 wt%) than XP2-1R2 (5 wt%); however, no explanation has been found for this result yet. Regarding the xerogels with various R_w_, the weight loss was not significantly different between the three ratios tested (around 4–6 wt% after 12 weeks). Finally, the gradual replacement of the SPU by TEOS in the xerogels architecture led to an increasing weight loss, as depicted in Figure 8. These results are mostly in good agreement with previously described hydrolytic degradation mechanisms of siloxanes [45]. With TEOS-based Si-O-Si networks being purely inorganic, their in vitro degradation in PBS solution led to the formation of a higher number of short hydrophilic silanol molecules, compared to the other xerogels. Thus, higher SPU by TEOS building blocks (from XP2-1R2 to XPT1-1) led to greater weight loss from the hydrolytic degradation of the inorganic Si-O-Si network.

Finally, the xerogel materials after 12 weeks of incubation were subjected to further analyses. Physical modifications of the surfaces observed without specific magnifications and zoomed pictures collected by SEM (magnification = ×500) are displayed in Supporting information (Figure SI.3). They were compared to controls corresponding to non-degraded xerogels. In the SEM pictures, strips from the mold surface are visible in the control and the samples. In most samples, one can clearly see the degradation, which is displayed through three main surface changes that can be observed in the pictures:

(1) The samples strips were eroded, resulting in flatter surfaces compared to the controls (in Series 2 and more specifically for XP2-1R8);
(2) The samples were eroded between the strips, resulting in more visible strips compared to the control (for XP6-1R2);
(3) The apparition of perforations along the surface (Series 2).

In Series 4, surface changes could not be observed on the samples as the control and the sample did not display flat surfaces nor strips. In this case, however, dispersed particle-like objects were present at the xerogels surface. These NPs could correspond to silica NPs (SiO_2_) obtained from the addition of TEOS, followed by the sol–gel process and drying, as previously described [23]. The phenomenon is especially highlighted for XPT1-1, where an equimolar TEOS/SPU ratio was used. Further analyses would be necessary to confirm this hypothesis.

## 4. Conclusions

Different renewable xerogels have been successfully prepared via sol–gel process from various biobased starting materials such as oligoPHB-diol and DDI to obtain materials with high biobased contents (until 78%) with a green approach. Based on previous studies, NCO terminated PUs were first prepared with various NCO/OH molar ratios and different diisocyanates (fossil-based HDI or biobased DDI). The organic–inorganic networks were then developed by modifying the NCO-terminated prepolymer onto siloxane precursor with APTMS as end-capper. Four series of xerogels were prepared with different architectures, in which different parameters were varied such as the water content or the gradual replacement of the PU-based precursor by TEOS, for instance. Consequently, the obtained hybrid organic–inorganic materials exhibited a large range of structures and properties. For instance, the inorganic crosslinking density increased with the NCO/OH ratio and decreased with R_w_, resulting in a higher hydrophobic character of the corresponding materials. Moreover, higher static mechanical properties than the corresponding pristine PU were obtained in the DDI-based xerogel. Finally, thanks to the siloxane network, hydrolytic degradation of the xerogels was significantly enhanced compared to equivalent organic materials.

As a perspective of this work and towards biomedical applications, biocompatibility, cytotoxicity and antibacterial activities should be assessed to confirm that these materials are highly promising candidates as scaffolds or wound dressing materials, for instance.

## Figures and Tables

**Table 1:** Compositions of the xerogels.

Xerogel	Series N°	Di-isocyanate	PU		SPU		Xerogels		Biobased Content [wt%]
			Name	NCO/OH Ratio	Name	APTMS Molar Eq.	R_w_	TEOS/SPU Ratio	
XP2-1R1	2	HDI	PUP2-1	2	SPU2-1	1	1	0	26
XP2-1R2	1, 2, 3 and 4	HDI	PUP2-1	2	SPU2-1	1	2	0	26
XP2-1R8	2	HDI	PUP2-1	2	SPU2-1	1	8	0	26
XP4-1R2	1	HDI	PUP4-1	4	SPU4-1	3	2	0	12
XP6-1R2	1	HDI	PUP6-1	6	SPU6-1	5	2	0	8
XPD2-1R2	3	DDI	PUPD2-1	2	SPUD2-1	1	2	0	78
XPT3-1	4	HDI	PUP2-1	2	SPU2-1	1	2	0.33	17
XPT1-1	4	HDI	PUP2-1	2	SPU2-1	1	2	1.00	13

**Table 2:** Main physico-chemical properties and parameters of the synthesized xerogels.

Xerogel	TGA T_5%_ [ °C]	TGA T_d1,max_ [ °C]	TGA T_d2,max_ [ °C]	TGA T_d3,max_ [ °C]	Char Residue at 650 °C [%]	DSC T_g_ [ °C]	DMA T_α_ [ °C]	*ν_e_* [mol.m^−3^]	*M_c_* [g.mol^−1^]
XP2-1R1	227	271	372	478	22.9	15	27	4900	301
XP2-1R2	242	271	375	476	19.1	18	22	4269	349
XP2-1R8	206	266	385	473	18.7	9	21	2680	612
XP4-1R2	209	261	389	482	31.8	11	18	1910	670
XP6-1R2	222	304	363	486	29.6	20	28	8508	170
XPD2-1R2	268	286	380	465	5.5	−14	−5	986	1617
XPT3-1	227	276	378	475	14.3	17	4	926	1382
XPT1-1	217	236	377	485	22.9	16	23	13234	118

## Data Availability

The data presented in this study are available on request from the corresponding author.

## Supplementary Materials

The following are available online at https://www.mdpi.com/article/10.3390/polym13234256/s1, Figure SI.1: ^1^H NMR of a) DDI, b) oligoPHB-diol and c) PUPD-2. Figure SI.2: Xerogels DSC thermograms. Figure SI.3: Pictures of the degraded xerogels after 12 weeks incubation without magnification (samples diameter = 1 cm) and zoomed observations by SEM (Magnification = ×500) compared to the corresponding controls. Table SI.1: Static mechanical properties of the xerogels. Scheme SI.1: Hydrolytic degradation mechanism of the siloxane network.
