# Supplementary material for: Synthesis of Biobased and Hybrid Polyurethane Xerogels from Bacterial Polyester for Potential Biomedical Applications
Source: Polymers (Basel). 2021 Dec 4;13(23):4256. doi: 10.3390/polym13234256 (PMC8659847; doi:10.3390/polym13234256)
Supplement: Supplementary file 1 [file polymers-13-04256-s001.zip › polymers-1489766-supplementary.pdf]

Supporting Information

Synthesis of biobased and hybrid polyurethane xerogels from bacterial polyester for potential biomedical applications

Sophie Wendels, Deyvid de Souza Porto, Luc Avérous*

BioTeam/ICPEES-ECPM, UMR CNRS 7515, University of Strasbourg, 25 rue Becquerel, Strasbourg Cedex 2, Strasbourg 67087, France

* Corresponding author : Prof. Luc Avérous, Phone : + 333 68852784, Fax : + 333 68852716, E-mail :luc.averous@unistra.fr

Figure SI.1. ^{1}H NMR of a) DDI, b) oligoPHB-diol and c) PUPD-2.

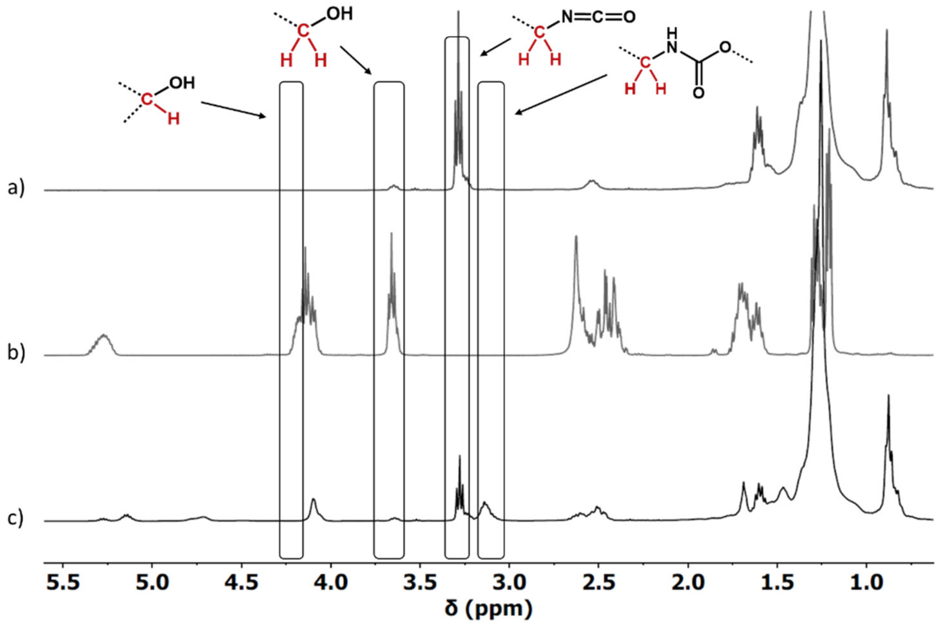

Figure SI.2. Xerogels DSC thermograms.

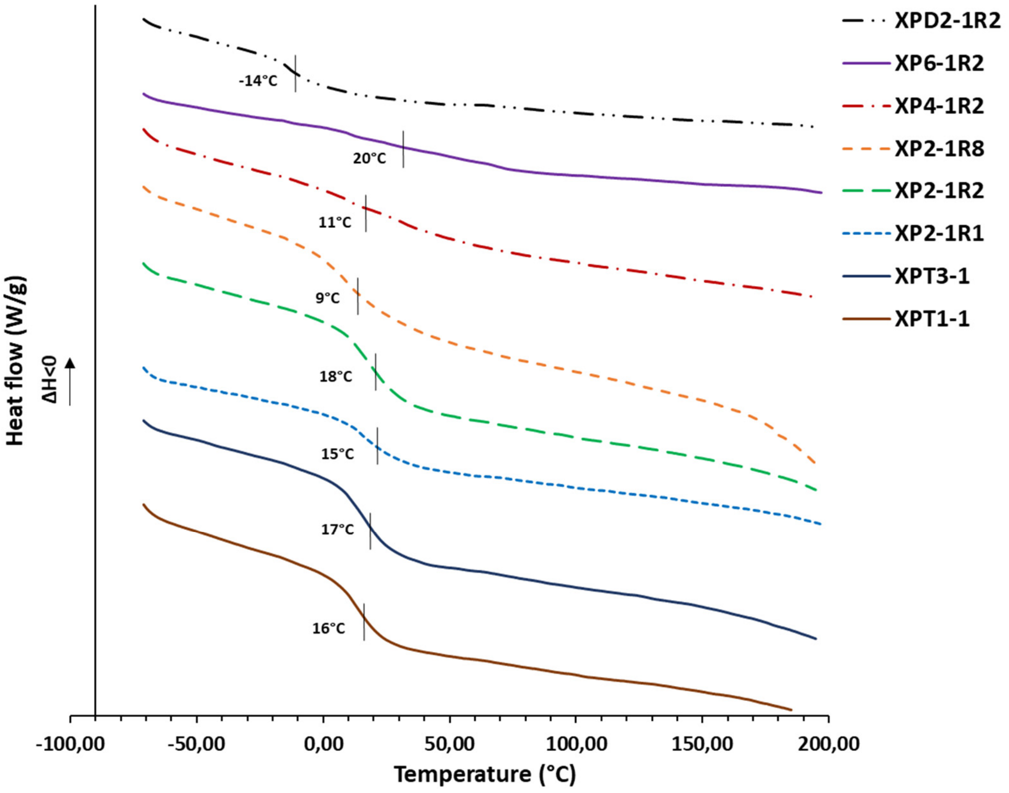

Table SI.1. Static mechanical properties of the xerogels.

Xerogel	Elastic modulus [MPa]	Tensile strength at break [MPa]	Elongation at break [%]
XP2-1R1	21.3 ± 1.6	6.0 ± 1.0	33 ± 3
XP2-1R2	20.6 ± 2.2	5.7 ± 0.8	31 ± 2
XP2-1R8	22.4 ± 1.4	3.0 ± 0.3	18 ± 2
XP4-1R2	79.0 ± 3.9	6.7 ± 0.2	15 ± 1
XP6-1R2	-	-	-
XPD2-1R2	7.4 ± 0.5	2.5 ± 0.3	37 ± 2
XPT3-1	15.4 ± 0.6	2.3 ± 0.9	16 ± 0.5
XPT1-1	18.9 ± 0.3	2.8 ± 0.5	16 ± 3

Scheme SI.1. Hydrolytic degradation mechanism of the siloxane network.

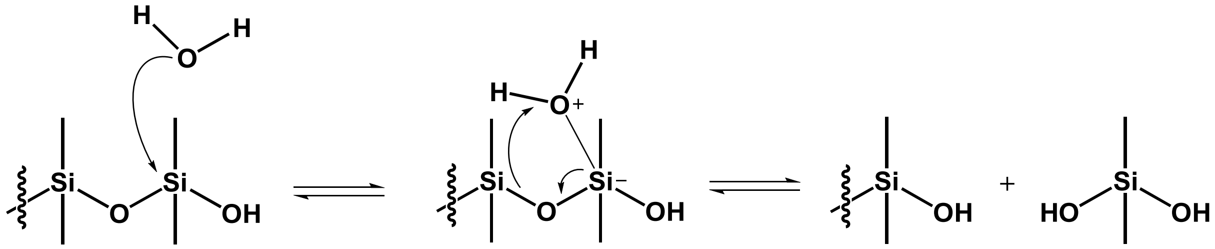

Figure SI.3. Pictures of the degraded xerogels after 12 weeks incubation without magnification (samples diameter = 1 cm) and zoomed observations by SEM (Magnification = x500) compared to the corresponding controls.

	Without magnification		SEM pictures	
	Control	Degraded sample	Control	Degraded sample
XP2-1R1	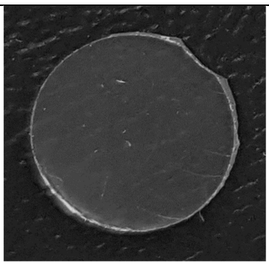	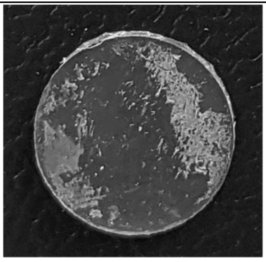	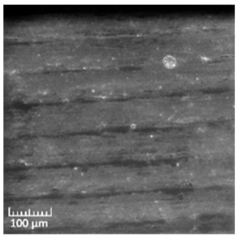	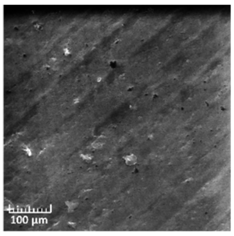
XP2-1R2	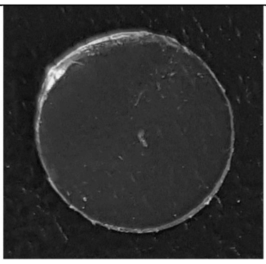	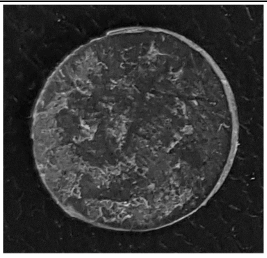	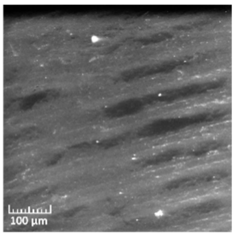	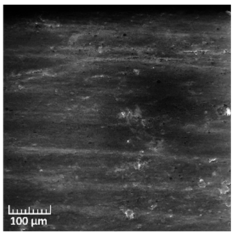
XP2-1R8	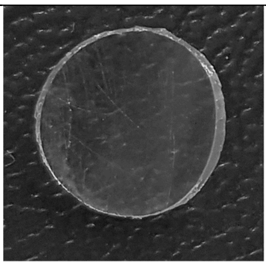	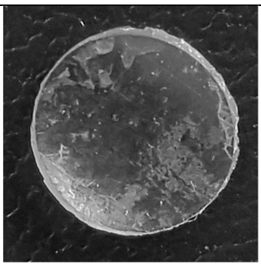	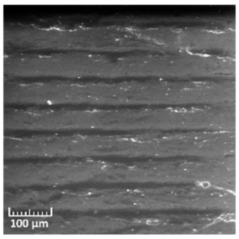	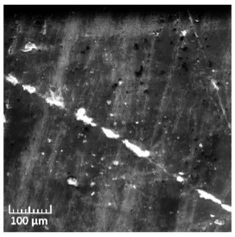
XP6-1R2	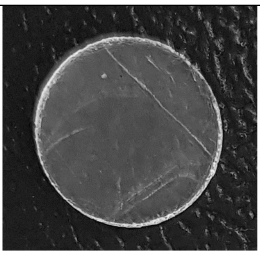	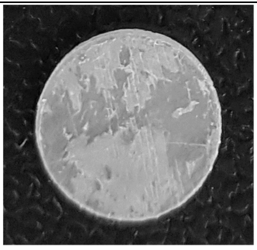	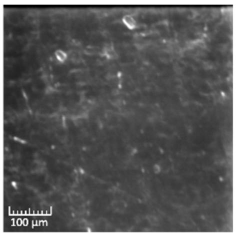	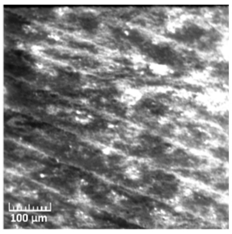
XPD 2-1R2	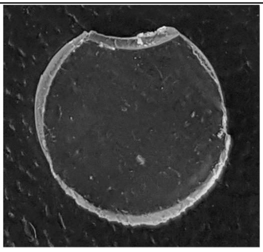	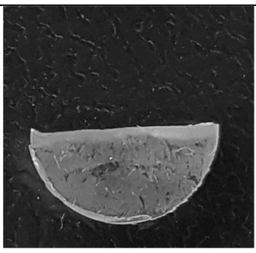	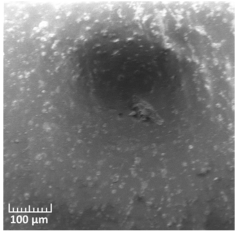	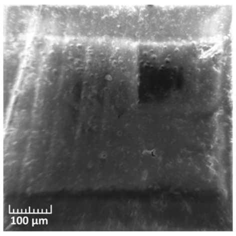
XPT1-1	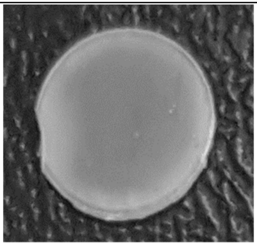	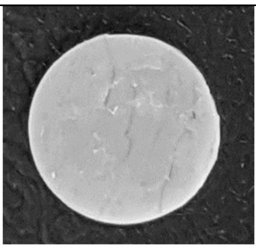	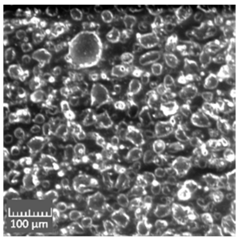	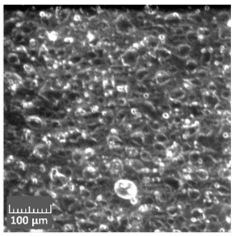
